# From Hive Sensors to Environmental DNA: Toward a Systems Biology Framework for Honeybee-Based Early Warning of Colony and Ecosystem Health

**DOI:** 10.3390/insects17070660

**Published:** 2026-06-24

**Authors:** Zunair Ahsan, Faouzi Haouala, Mokhtar Rejili

**Affiliations:** 1 College of Animal Science and Technology, Yangzhou University, 88 South University Rd, Yangzhou 225009, China; mh24057@stu.yzu.edu.cn; 2 Department of Biology, College of Sciences, Imam Mohammad Ibn Saud Islamic University (IMSIU), Riyadh 11623, Saudi Arabia; fmhaouala@imamu.edu.sa

**Keywords:** *Apis mellifera*, precision beekeeping, environmental DNA (eDNA), biosentinel, early warning system, colony health, exposomics, systems biology

## Abstract

Honeybees (*Apis mellifera*) serve as biological sentinels because their foraging behavior links colony health to environmental conditions. Traditional hive inspections are invasive, observer-dependent, and often detect problems only after symptoms appear. This review synthesizes advances in precision beekeeping, environmental DNA (eDNA) metabarcoding, exposomics, and artificial intelligence to propose the Honeybee-Based Early Warning System (H-BEWS), a unified framework that integrates digital sensors, molecular and chemical monitoring, and ecological data into a predictive early warning system for both colony and ecosystem health. By linking anomalies detected by hive sensors to targeted molecular and chemical analyses, H-BEWS enables proactive interventions and environmental surveillance, supporting a One Health perspective. Unlike previous reviews that focus on individual technologies, H-BEWS emphasizes multi-layered integration, predictive risk assessment, and ecosystem-level insights, providing a novel conceptual framework for early detection of colony stress and environmental hazards. The approach offers practical applications for beekeepers, researchers, and policymakers by converting real-time data into actionable insights and informing management decisions. Challenges include sensor standardization, data integration, AI validation, and equitable access for small-scale beekeepers. Future directions will focus on real-time sequencing, multimodal AI models, digital twin creation, and the development of global surveillance networks. H-BEWS demonstrates how an integrative, multi-layered approach can transform honeybee colonies into living biosensors, providing actionable insights for both apiculture management and ecosystem monitoring.

## Simple Summary

In addition to producing honey, honeybees are live sensors that continuously take in their surroundings. They come across flowers, pollutants, illnesses, and other biological things while foraging throughout landscapes, all of which leave traces inside the hive. Scientists can identify early indicators of colony stress before bees perish or vanish by using sensors to monitor hive weight, temperature, humidity, sounds, and flight activity, as well as by analyzing DNA from honey, hive debris, and pollen. This review explores how integrating digital sensors, environmental DNA, chemical residue analysis, and artificial intelligence can turn honeybee hives into early warning systems for both colony collapse and broader ecosystem health. H-BEWS (Honeybee-Based Early Warning System) is a suggested framework that could assist beekeepers in taking action before colonies fail and notify regulators of environmental hazards such pesticide contamination, developing diseases, or loss of floral resources.

## 1. Introduction

### 1.1. Honeybees as Sentinel Pollinators: Limits of Traditional Monitoring

Because their colony success reflects both internal biological condition and exterior landscape quality, honeybees occupy a unique position between agriculture and the wider environment. In addition to supporting crop productivity, food security, and ecosystem functioning as regulated pollinators, *Apis mellifera* colonies’ extensive foraging range enables them to come into contact with floral resources, contaminants, diseases, and other biological materials across diverse landscapes [1]. Because of their dual function, honeybees are useful as both pollinators and biological sentinels that may detect shifts in the health of the agroecosystem.

Honeybees’ mobility, social organization, and frequent contact with soil, water, air, vegetation, pollen, nectar, and anthropogenic toxins make them an excellent choice for early-warning monitoring. Bees gather materials from vast surrounding areas and can provide information on both organic and inorganic pollutants, honeybees and hive products as useful biomonitoring tools [2]. According to Bromenshenk et al. [3], bees can act as biosensors because of their chemosensory skills and foraging habits, which enable them to recognize and react to chemical cues in their surroundings. Although it has significant limits for early warning, traditional hive inspection is still crucial. Visual inspection-based colony assessment is time-consuming, observer-dependent, disruptive, and frequently finds issues only after symptoms become apparent. Traditional visual evaluations can be subject to observer bias; hence, in order to increase consistency, new quantitative methods utilizing adult bee weight, brood imaging, and image analysis have been suggested [4]. The need for standardized instruments that evaluate several aspects of colony health, such as queen performance, demographics, hive products, disease, infection, infestation, management techniques, and environmental factors, was also emphasized by [5].

Several shortcomings of human inspection are addressed by remote and sensor-based monitoring, which produces continuous, non-invasive, and colony-specific data. For example, Janota et al. [6] developed a non-invasive robotic comb mapping system to track brood development and colony structure, demonstrating advanced automated monitoring. Milovanović et al. [7] evaluated integrated non-invasive sensor arrays, showing that multimodal sensor fusion improves real-time colony assessment. Ahumada-García et al. [8] applied deep learning to hive acoustic data for real-time anomaly detection, highlighting predictive applications for early warning systems. Precision beekeeping has concentrated on both exterior factors like rainfall, wind, and ambient temperature as well as inside-hive characteristics like weight, temperature, humidity, flight activity, sound, vibration, and gases [9]. By connecting environmental variance with honey production patterns, Catania and Vallone showed how weather monitoring, temperature, humidity, and hive weight may help beekeepers make decisions [10]. Colony strength may be linked to hive temperature data, suggesting that sensor-derived signals can represent biologically significant colony states [11]. Within the H-BEWS framework, digital sensors act as the first predictive layer, providing continuous, real-time measurements of colony weight, temperature, humidity, flight activity, CO_2_, sound, and vibration. These sensor-derived anomalies can serve as early warning signals that trigger molecular and chemical analyses, enabling a predictive, rather than reactive, approach to colony and ecosystem monitoring. By integrating sensor data streams into H-BEWS, beekeepers and researchers can detect subtle changes in colony health or environmental stressors before visible symptoms occur. This integration allows for timely interventions, supports decision-making, and links internal hive dynamics with landscape-level environmental conditions, fulfilling the multi-layer vision of H-BEWS [12,13].

This early-warning potential extends beyond colony physiology through molecular techniques. Bees, plants, bacteria, pests, and viruses can leave biological traces in honey, hive detritus, pollen, and other hive matrices. Multi-kingdom environmental DNA signals, such as those from arthropods, plants, fungi, bacteria, and viruses, may be recovered using shotgun metagenomics of honey DNA [14]. Hill et al. [15] demonstrated that surface swab sampling at hive entrances outperforms traditional hive matrices for recovering eDNA and eRNA, improving detection of colony and environmental signals. Within the H-BEWS framework, molecular phenotyping using eDNA acts as a confirmatory and interpretive layer. When digital sensors detect anomalies in hive conditions, targeted eDNA analyses can identify pathogens, pests, or shifts in microbial and plant communities, enabling timely interventions. By combining real-time sensor signals with molecular data, H-BEWS transforms isolated observations into predictive insights, allowing both colony health and environmental stressors to be monitored concurrently. This integrative approach ensures that early warning alerts are not only sensitive to immediate changes but also informative about the underlying biological causes, supporting more accurate decision-making and enhancing the predictive power of the system [16,17]. Henneken et al. [18] showed that eDNA metabarcoding of hive detritus may identify bacterial, fungal, and arthropod populations, including diseases, pests, beneficial species, and taxa that are significant to agriculture. Honey-based biosurveys are an emerging method for tracking biodiversity and comprehending ecological interactions captured by honeybee foraging behavior [19].

When taken as a whole, these investigations facilitate the shift from delayed, visual colony diagnosis to integrated early warning systems that incorporate molecular evidence from materials derived from hives with colony-level sensor data. Because fodder availability, pathogen pressure, pollution, landscape composition, and management circumstances all influence colony health and ecosystem health, this integration is crucial. Therefore, in a more comprehensive systems biology context, honeybees offer a scientifically supported model for connecting digital hive monitoring with environmental DNA surveillance. Despite the rapid expansion of technological approaches for monitoring honeybee colonies, there is currently no unified framework that systematically integrates digital, molecular, chemical, and ecological data to support predictive, early-warning assessment of colony and ecosystem health. Previous reviews have tended to focus individually on specific classes of monitoring tools (e.g., continuous hive sensors, smart hive technologies, or IoT-based systems) without articulating how these modalities can be combined into a comprehensive interpretive system for both apicultural and environmental applications. In contrast, this review proposes the Honeybee-Based Early Warning System (H-BEWS) as an integrative conceptual framework that not only captures multi-layered phenotypic signals from honeybee activity and hive matrices, but also links these data streams to actionable indicators within a One Health context. By emphasizing convergence of modalities and their potential for predictive analytics, the H-BEWS framework aims to guide future research, standardization efforts, and practical implementations that transcend traditional, reactive colony diagnostics.

Honeybee colony health is intrinsically connected to ecosystem integrity and, by extension, human wellbeing. Because they provide critical pollination services that support agricultural productivity and food security, changes in honeybee health can reflect broader risks to ecosystem functioning and human health [20]. Honeybees also serve as effective biological sentinels for environmental stressors, including chemical pollutants and pathogen exposure, making them valuable within a One Health framework that recognizes the interconnectedness of human, animal, and environmental health [21,22]. By integrating digital, molecular, and ecological data into the H-BEWS framework, this review situates honeybee monitoring as a component of early warning systems capable of anticipating hazards that affect both pollinators and broader public health outcomes.

### 1.2. From Reactive to Predictive: The Hive as an Integrated Biosensor

Before colony loss is apparent, honeybee colonies might reveal new threats.

Because apparent symptoms typically arise after interacting forces have already impacted colony function, reactive colony health management is frequently inadequate. According to Hristov et al. [23] single-factor diagnosis is erroneous since colony losses are influenced by a variety of factors, such as parasites, diseases, management, climate, agricultural practices, and pesticides. The connection between colony loss and nutritional deficiencies, *Varroa destructor*, mixed viral infections, bacterial and fungal diseases, and apiary management supports the necessity of routine monitoring prior to collapse-level damage [24].

Colony conditions may now be tracked using quantifiable internal and external cues, making predictive management more practical. Temperature, relative humidity, weight, and meteorological data can be utilized in predictive models to detect anomalous hive conditions and aid in decision-making, as demonstrated by [25]. Brood levels, pollen, honey, *Varroa destructor*, deformed wing virus, and bee population were found to be significant predictors of colony survival using machine learning techniques on colony loss data [26]. Šerba et al. [27] applied machine learning to honeybee flight dynamics, demonstrating that subtle changes in foraging behavior can predict colony stress, which supports the predictive layer in H-BEWS. Within the H-BEWS framework, AI and machine learning serve as the decision-making layer, integrating digital sensor streams, molecular signals, chemical analyses, and ecological data to generate predictive insights about colony and ecosystem health. By combining multi-layered inputs, AI models can detect anomalies and forecast potential stressors before observable symptoms appear, enabling proactive interventions. This integration transforms H-BEWS into a predictive early warning system, where AI not only identifies risks but also contextualizes them across environmental and management factors, supporting evidence-based decisions within a One Health perspective. Recent advances in multimodal AI, including data fusion and predictive modeling, demonstrate the feasibility of using integrated colony monitoring data to anticipate pathogen outbreaks, nutritional stress, and environmental hazards [17,28].

Because foragers constantly gather chemical and biological traces from the surrounding environment, the hive can also function as a living sampling tool. Honey can preserve molecular evidence of both colony-associated and environmental sources, as honey DNA contains multi-kingdom signals from honeybees, plants, fungi, bacteria, viruses, and other arthropods [14]. Additionally, Hristov et al. [18] demonstrated that hive debris eDNA metabarcoding can identify groups of arthropods, fungi, and bacteria, including pests, diseases, beneficial species, and taxa that are significant to agriculture.

This sampling capacity is further enhanced by chemical and ecological signals. Beehive colonies were impacted by repeated exposure to diesel exhaust, including changes in colony weight, suggesting that air pollution can alter quantifiable colony-level outcomes [29]. Portable membrane inlet mass spectrometry can identify contaminants and volatile chemicals in hive air, supporting the use of hive atmosphere as an environmental contamination indicator [30]. In H-BEWS, chemical phenotyping provides an additional predictive layer, detecting pesticides, acaricides, heavy metals, and volatile organic compounds that can compromise colony and ecosystem health. When anomalies are observed in digital sensors or molecular analyses, chemical data can help pinpoint the specific environmental stressor, enabling targeted management actions. Integrating chemical monitoring into H-BEWS ensures that alerts are not only timely but also actionable, linking contamination events to potential effects on colony performance and surrounding ecosystems. This multi-layered approach reinforces H-BEWS as a predictive, decision-support system that moves beyond reactive hive assessments [28,31]. In support of their function in ambient bioindication, honeybee colonies may also gather airborne biological material at hive entrances [32]. Uthoff et al. [33] demonstrated that combining multiple sensor streams with landscape context can improve assessments of hive strength and foraging efficiency, reinforcing the value of integrated monitoring for early warning. Within the H-BEWS framework, ecological phenotyping provides essential context that links colony responses to landscape and environmental conditions. Landscape features such as land use, vegetation cover, and forage availability influence honeybee foraging patterns, colony nutrition, and seasonal resource access, and these ecological variables can modulate sensor and molecular signals captured by hive monitoring. For example, colony monitoring devices have been used to relate digital hive data to differences in land use and forage quality, demonstrating how static and dynamic landscape features can predict hive weight changes and colony conditions across agricultural gradients [34]. Similarly, temporal variation in pollen resource use by honeybees reflects ecological shifts in floral availability throughout the season, linking landscape phenology with colony nutritional ecology [35]. By integrating ecological data streams into H-BEWS alongside digital, molecular, and chemical signals, the system can better anticipate environmental pressures on pollinator health and provide early warning insights relevant to both apiculture and ecosystem sustainability.

**Figure 1:**
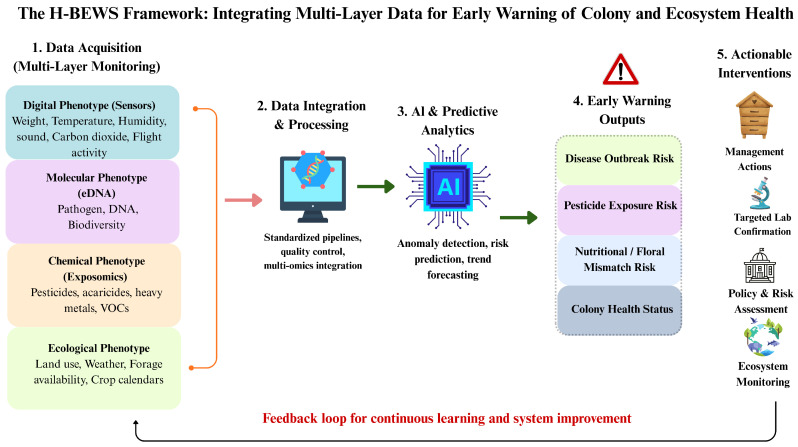
Conceptual framework of the Honeybee-Based Early Warning System (H-BEWS), illustrating integration of digital, molecular, chemical, and ecological layers, AI-based data integration, and actionable outputs for colony, ecosystem, and human health.

The literature is still disjointed despite these developments. Pollution studies mostly describe chemical exposure, while eDNA studies primarily describe molecular detection, and sensor-based studies primarily provide digital indicators of colony state. According to de Jongh et al. [21], interdisciplinary approaches that connect honeybees, contaminants, climate-related stresses, and One Health outcomes are necessary. This gap facilitates the creation of a single predictive framework for colony and ecosystem health that integrates digital, molecular, chemical, and ecological data. Honeybee monitoring can transition from late diagnosis to early warning with this kind of integration. The Honeybee-Based Early Warning System (H-BEWS) integrates multi-layer colony monitoring into a predictive framework, as shown in Figure 1.

## 2. Historical Evolution of Honeybee Monitoring

### 2.1. Classical Monitoring

Because visual examination enables beekeepers and researchers to examine adult bee population, brood area, food reserves, queen presence, and obvious symptoms of pests or disease, it has remained the fundamental tool for assessing colony state. Directly measurable field characteristics of managed honeybee health include queen presence and performance, colony demography, in-hive products, and disease or infestation [5]. Although these measurements offer a useful basis for colony evaluation, their precision is dependent on the biological condition of the colony at the time of observation, the experience of the observer, and the timing of the inspection.

Although it is not always easy to understand, brood pattern has long been employed as a gauge of queen performance and colony output. Recent standardized approaches for assessing classical colony parameters including strength indicators such as adult bee population, comb construction, and flight activity have been proposed to improve comparability across studies and management contexts [36]. When queens from low-brood colonies were transferred to healthier colony conditions, brood pattern might improve and the bad brood pattern was not consistently linked to queen quality parameters. This suggests that brood appearance may be influenced by environmental factors or colony-level factors rather than queen failure alone. Groeneveld et al. [37] later showed through simulation that brood-related indicators, especially capped brood, may provide earlier warning signals of colony stress than adult bee abundance or honey reserves alone.

Adult bee strength is another central element of classical monitoring because it reflects worker population size, colony labor capacity, thermoregulation potential, and readiness for pollination services. Using measures of colony strength in England and Wales, it is found that adult bees in weak colonies carried more diseases, with deformed wing virus demonstrating predictive value for lower colony strength [38]. Connection between overwintering survival and brood production, pollen collecting, and *Varroa destructor* levels demonstrates that traditional colony metrics can have predictive biological significance when evaluated collectively [39].

**Figure 2:**
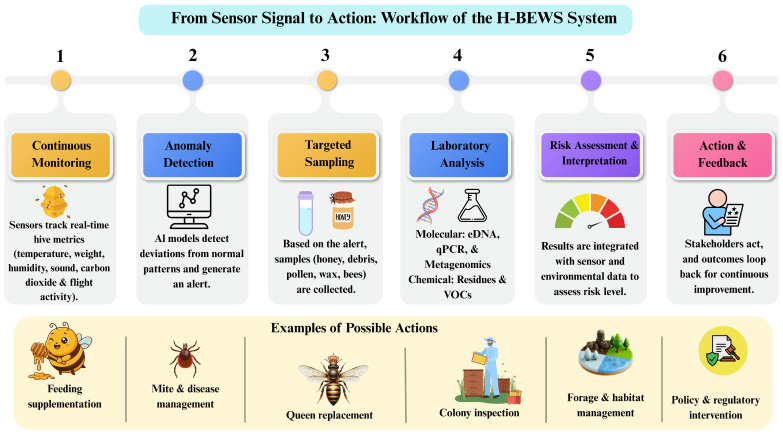
Comparison of honeybee colony monitoring modalities, illustrating temporal resolution, invasiveness, key parameters, and limitations for classical, molecular, metagenomic, digital, and eDNA-based monitoring approaches.

Because honey and pollen reserves indicate both colony readiness and the availability of landscape resources, food stores continue to be a major inspection target. Pesticide exposure may be linked to queen failure, broodless periods, weaker colonies, and decreased overwintering survival, according to Dively et al. [40], who incorporated honey stores and other colony performance indicators in a long-term colony health evaluation. In another study, Varroa infestation and deformed wing virus were adversely correlated with colony strength: weak colonies were linked to environments less conducive to bee nutrition [41]. Different monitoring modalities vary in temporal resolution, invasiveness, and key parameters Figure 2.

Because mite pressure is directly associated with viral transmission and colony collapse, pest counts, particularly *Varroa destructor* monitoring, are crucial to traditional colony evaluation. Colonies chosen for low mite population expansion had lower levels of viral infection than colonies with strong Varroa population growth [42]. The ongoing significance of direct pest evaluation during routine inspection is supported by these findings.

Because queen presence, egg laying, brood continuity, and colony organization affect population renewal, queen status is also essential to traditional monitoring. However, evaluating queens only on the basis of obvious colony symptoms may be deceptive. Queen introduction success, colony size, honey yield, and winter survival are pertinent metrics when assessing queen performance after overwintering [43], brood pattern alone may not be a reliable indicator of queen quality [44]. The limitations of classical examination include subjectivity, invasiveness, labor demand, and sporadic sampling, despite the fact that it offers direct biological information. Objective measurements using hive weights, frame photos, internal temperature, and sensor records have been suggested after observing that standard visual assessment may not be adequate for identifying subtle colony-level effects, especially under sublethal pesticide exposure [45]. There is a need for more uniform quantitative methodologies because current colony evaluation techniques rely on subjective, observer-dependent visual estimations [4]. Although it only offers sporadic glimpses of a dynamic superorganism, classical monitoring is nevertheless essential.

### 2.2. Molecular Era

Molecular techniques replaced symptom-based observation in honeybee monitoring with direct biological agent detection.

Because they enable the precise identification of bacterial, fungal, microsporidian, and viral targets before a diagnosis can be made based solely on colony appearance, PCR and RT-PCR have become essential methods for identifying honeybee pathogens. Grabensteiner et al. [46] demonstrated that several commercially significant honeybee viruses could be quickly identified from bee specimens by developing a multiplex RT-PCR technique for simultaneous identification of acute bee paralysis virus, black queen cell virus, and sacbrood virus. Genetic screening of adult bees and brood in England and Wales discovered that diseases were more prevalent in poor colonies, with deformed wing virus exhibiting a negative correlation with colony strength [38].

Because honeybee viruses frequently interact with *Varroa destructor* and may be difficult to detect through outward indications until colony destruction is advanced, viral surveillance became particularly crucial. Injected viral RNA may proliferate and cause chronic paralysis symptoms in adult bees by creating an experimental infection model for the chronic bee paralysis virus [47]. *Apis mellifera solinvivirus 1*, a novel honeybee RNA virus that had gone unnoticed for years and was discovered in archived apiary samples throughout the United States using metagenomic analysis [48]. Several pathogenic RNA viruses, such as deformed wing virus, acute bee paralysis virus, black queen cell virus, and sacbrood virus, were detected using next-generation sequencing on honeybee subspecies and related Varroa mites from the Middle East and North Africa [49].

By making it possible to identify bee lineages, floral sources, and biological material found in hive products, DNA barcoding and related marker-based techniques extended molecular surveillance beyond disease detection. In order to connect population genetics with pathogen surveillance, *Nosema ceranae* and *Nosema apis* infection were compared across wild and controlled *Apis mellifera* colonies using mitochondrial DNA haplotypes [50]. By using eDNA metabarcoding to identify bacterial, floral, and honeybee DNA signatures in commercial honey, honey may be used to determine floral and geographical origin [51]. Molecular plant identification in honey-based ecological tracing using ITS2 sequence data from honey eDNA is important for identifying plant species linked to honey produced by *Apis dorsata* and stingless bees [52].

By enabling the untargeted discovery of numerous organisms in a single analysis, metagenomics expanded the discipline. Honey might maintain molecular information from the colony and its surroundings by using shotgun metagenomics of honey DNA to recover a multi-kingdom signature that includes arthropods, plants, fungi, bacteria, and viruses [14]. *Melissococcus plutonius*, *Paenibacillus larvae*, *Varroa destructor*, *Aethina tumida*, *Vairimorpha ceranae*, and several honeybee viruses were among the pests and pathogens that [53] identified using an integrated eDNA and eRNA meta-omics approach on royal jelly. They also identified beneficial bacterial groups [53].

Environmental DNA metabarcoding significantly changed monitoring to focus on community-level, non-invasive surveillance by using eDNA metabarcoding on hive debris, bacterial, fungal, and arthropod communities that included diseases, beneficial species, hive pests, and agriculturally significant taxa [18]. Honey can serve as a reservoir for pathogen surveillance and detection of *Spiroplasma apis* and *Spiroplasma melliferum* in Australian honey samples using honey eDNA and pathogen-specific PCR [54]. These methods demonstrate the ability of molecular monitoring to identify creatures and interactions that are invisible to the naked eye. Therefore, the biological clarity required to link colony health to pathogen pressure, floral use, genetic identity, and environmental exposure is provided by the molecular era.

### 2.3. Digital Era

Periodic inspection was replaced by ongoing colony observation in honeybee assessment thanks to digital monitoring. Beekeepers may remotely monitor hive conditions using sensors that detect temperature, humidity, weight, sound, gas concentration, flight activity, and external weather thanks to smart hives and Internet of Things-based devices. Precision beekeeping, is the process of connecting hive measurements with biological colony conditions via IoT infrastructure, sensor networks, communication systems, power sources, and data analysis [55]. Cecchi et al. [56] created a multisensory platform for real-time beehive monitoring and demonstrated how information on colony state and its interactions with the environment can be obtained by combining hive weight, sound, temperature, humidity, carbon dioxide, and weather data.

Precision apiculture has grown most rapidly around quantifiable hive variables that can be collected on a regular basis without having to open the colony. Precision beekeeping research is primarily organized around internal hive parameters like weight, temperature, relative humidity, flight activity, sound, vibration, and gases, as well as external variables like wind, rainfall, and ambient temperature [9].

Commercial and experimental accuracy beekeeping systems often use wireless sensor networks and remote data platforms to monitor weight, temperature, humidity, sound, gasses, flight activity, and swarming-related characteristics [16]. Because colony noises reveal information about internal activity, communication, and disruption, acoustic monitoring has emerged as one of the most promising non-invasive methods. Hive noises can be utilized to identify changes in colony state utilizing microphones and acquisition systems, as demonstrated by assessment of honeybee sound analysis techniques by Terenzi et al. [57]. In their study of automated beehive acoustics, there is a need for improved reproducibility, transparent experimental setup descriptions, and open datasets. They also identified sound analysis as a developing field for remote hive monitoring [58]. According to Sharif et al., acoustic monitoring for swarming, honeybee health, pesticide effects, and environmental pollution at apiaries is supported by expert assessment [59].

Digital hive evaluation is enhanced by thermal and image-based techniques that provide physiological and visual data. Sensor placement affects the accuracy of temperature-based strength prediction and hive temperature data may be connected to colony strength [11]. According to Marchal et al. [60], automated movement tracking in conjunction with connected hives can produce long-term behavioral data from both inside and outside the hive, facilitating the advancement of computational methods for studying bee behavior. These techniques enable the recording of colony dynamics at a better temporal resolution and lessen reliance on manual observation.

The digital age has moved from monitoring to prediction thanks to AI-assisted solutions. In order to model bee entrance and exit behavior in connection to environmental variables, Andrijević et al. [61] created an Internet of Things monitoring and prediction system using hive sensors, bee counters, cloud storage, recurrent neural networks, and alarms. Through AI applications in apiculture, machine learning techniques can enhance hive management, health monitoring, pest and disease identification, and climate-related decision-making by utilizing sound, pictures, and sensor readings [62]. Additionally, deep learning, computer vision, and multimodal sensor fusion are becoming increasingly popular in smart beehive technologies, especially for illness detection and colony behavior modeling [13]. Adoption of these solutions is dependent on cost and usability in addition to technical performance. According to a survey conducted by [63] among beekeepers in France, Germany, and Greece, 45% of them utilized some kind of precision apiculture equipment, most frequently hive scales. However, the expensive cost of these systems continues to be a significant obstacle to their wider usage. The need for shared data ecosystems in apiculture is emphasized, as varied datasets from hive weight, temperature, images, videos, audio, queen status, pests, and activity present standardization challenges for machine learning applications [64].

The continuous data layer required for early-warning honeybee monitoring is made available by the digital age.

## 3. Hive Sensors as Real-Time Physiological Signals of the Colony

### 3.1. Hive Weight

Because hive weight represents the cumulative effects of nectar intake, honey storage, bee traffic, food consumption, and abrupt colony-level events, it is one of the most direct real-time indicators of colony activity. By observing these changes without opening the hive, continuous weighing minimizes disturbance and provides repeated measurements that are not possible with traditional inspection [12].

As returning foragers carry nectar, which is subsequently processed and stored as honey, hive weight usually increases during nectar flow. Hive scales are useful for determining the intensity of foraging and the availability of resources in the surrounding landscape because [65] demonstrated that within-day hive weight patterns can distinguish between morning weight loss caused by departing foragers and afternoon weight gain linked to returning foragers. Weight monitoring can aid in the evaluation of honey production, with daily production declines associated with external weather stressors like wind and lower temperatures [10].

Forager traffic can also be inferred from short-term weight variations. Although the strength and direction of the relationship vary among colonies, camera-based bee traffic can be related within the same day. This indicates that weight data are most informative when interpreted with colony-specific and time-scale-aware models [66]. In order to promote scale data’s usage as an early warning signal for weakening colonies, Arias-Calluari et al. [67] modeled daily hive weight variation and demonstrated that it can estimate biologically significant indicators like food collected and the number of active foragers.

Hive weight can also be used to identify sudden occurrences. Continuous weight and temperature records, according to Meikle et al. [12], can differentiate between substantial disruptions, such as patterns linked to adult bee loss and swarming, and regular colony phenology. Remote swarming detection offers practical utility, because prompt notifications can minimize financial losses if beekeepers react promptly [68]. IoT-based hive monitoring might aid in the prediction of honey stealing, a detrimental occurrence associated with the loss of resources and the spread of disease among colonies [69].

Overall, when combined with environmental and behavioral data, hive weight monitoring transforms changes in colony mass into a non-invasive physiological indicator of nectar flow, honey production, foraging activity, swarming, robbing, and colony decline.

### 3.2. Temperature

Because brood growth depends on stable thermal settings and because loss of thermal control can imply weakness before visible outward signs manifest, temperature is a key physiological indication of the honeybee colony. Colony longevity depends on having enough bees readily accessible for heating activities, and in-hive workers maintaining brood nest heating [70]. The effectiveness of brood thermoregulation is more strongly correlated with the quantity of brood than with the total number of adult bees, suggesting that temperature stability is a better indicator of active brood-rearing status than colony size alone [71].

Colony strength can also be estimated using the internal temperature of the hive. Hives with live bees kept higher and more stable temperatures than empty hives. They also discovered that the mean hive temperature increased with each additional frame of bees, with the central sensor location providing the strongest correlation with colony strength [11]. Colony phenology and post-winter conditions can be inferred from average internal temperature and within-day temperature fluctuation, with weaker colonies exhibiting less temperature control [72].

Particularly useful for identifying stress and brood status are temperature trends. Meikle et al. demonstrated that temperature variability is inversely correlated with brood mass, suggesting that unstable temperature may indicate decreased brood presence or poor brood care [73]. They also demonstrated that continuous temperature data may be utilized in conjunction with weight records to monitor colony phenology. Temperature sensing can be used as an early warning system for colony failure since winter temperature patterns can be used to predict colony mortality and disclose brood condition [74].

When combined with other hive signals, temperature sensors may also help determine the risk of swarming. Continuous surveillance of temperature, weight, humidity, gasses, vibration, sound, and traffic can yield non-invasive data on phenology and colony behavior, according to [12]. In addition to weight, humidity, flight activity, sound, vibration, gasses, and external weather data, Alleri et al., [9] recognized interior temperature as one of the primary hive characteristics evaluated in precision beekeeping systems. Thus, temperature monitoring offers a brief, non-invasive indication of brood thermoregulation, colony strength, change related to swarming, and brood loss associated with stress.

### 3.3. Humidity, Sound, and Vibration

Because it is associated with nectar dehydration, brood care, and interior circumstances that can either promote or limit biological stress, humidity is a crucial hive indicator. Relative humidity as part of ongoing colony condition monitoring is useful when integrated with temperature, sound, CO_2_, weight, and external weather factors in a real-time multisensory hive platform [75]. In order to promote the use of distributed humidity sensing rather than single-point measurement for remote hive assessment, ref. [76] additionally tracked relative humidity at many internal hive points.

Humidity has direct biological relevance for brood development because eggs and immature stages are sensitive to the hive microclimate. Humidity can be combined with colony-level characteristics in predictive monitoring datasets by gathering continuous relative humidity, temperature, and audio data from 53 colonies along with expert phenotypic measures, such as bee population, brood cells, Varroa infestation, honey yield, queen status, and winter mortality [77]. Under measured in-hive temperature and relative humidity conditions, the strong colonies had higher forager movement, larger honey and pollen areas, and greater egg, larval, and sealed brood areas. This suggests that humidity data, when combined with brood and foraging measures, can help interpret colony strength [78].

Because nectar needs to be dried in order to form stable honey, humidity and honey ripening are related. Mitchell et al. [79] linked humidity dynamics to the physical process of nectar-to-honey conversion by describing how nectar water content, honey production rate, entrance size, and nest thermal conductance affect nest humidity. Internal hive humidity varied between landscapes and honeybee haplotypes, and colony weight increase also varied [80]. This suggests that humidity, thermoregulation, and productivity are related to both environment and colony biology.

Humidity should be interpreted cautiously when it comes to disease, however the research that is now available indicates that humidity can influence the risk of pathogens and parasites. High rainfall, high relative humidity, and high hive humidity were linked to more pest and disease issues in tropical honeybee colonies [81]. Under certain nest settings, increasing nest humidity may decrease *Varroa destructor* fertility, demonstrating how humidity can affect disease ecology in both detrimental and possibly beneficial ways depending on the situation [79].

Because sound and vibration record activity produced by bees themselves, they offer behavioral indications that complement temperature and humidity. After reviewing hive sound analysis, Terenzi et al. [57] came to the conclusion that since honeybees utilize sound to communicate within the hive, acoustic monitoring can disclose colony status and abrupt changes. In order to corroborate the acoustic detection of queenlessness, the removal of the queen resulted in noticeable changes in sound within an hour, with signals becoming more intense over the next several hours and reverting to normal after the queen was reintroduced [82].

Swarming and brood growth are two areas where vibration sense is especially helpful. In order to monitor brood-related colony development with fewer invasive inspections, Bencsik et al. [83] discovered a good correlation between accelerometer-based vibrational measures and the brood cycle close to the sensor. In addition to recording queen piping patterns during the swarming season, ref. [84] used vibrational spectra and machine learning algorithms to accurately forecast swarming prior to the occurrence. A mild artificial vibrational pulse can measure the movement and restfulness of colonies, and this response can be used to identify queenless colonies [85]. When combined, humidity, sound, and vibration sensors allow for real-time interpretation of brood care, honey processing, disease-favorable circumstances, queen status, swarming risk, disturbance, and colony activity in addition to static environmental data.

### 3.4. Gas Sensing, Flight Activity, and Computer Vision

Because CO_2_, respiration-linked gases, and volatile compounds can vary with colony size, ventilation, brood activity, food processing, contamination, and disease-related stress, gas sensors provide a metabolic and chemical layer to hive monitoring. Along with weight, temperature, humidity, sound, vibration, and forager traffic, breathing gasses are a component of ongoing honeybee colony monitoring [12]. Gas sensing may be integrated with physical and behavioral signals for non-invasive hive assessment by integrating CO_2_ with temperature, humidity, sound, weight, and meteorological factors in a real-time multisensor hive platform [75]. Because CO_2_ represents the ventilation and respiration patterns of colonies, it is particularly helpful. Even when hive ventilation was changed, honeybee colonies maintained robust daily CO_2_ cycles with maximum concentrations reaching 11,000 ppm, suggesting that CO_2_ control is an active colony-level mechanism [86].

A CO_2_ monitoring system for honeybee hives was created by [87], who found that variations in measured CO_2_ could be used to estimate colony size and that CO_2_ decreased over many weeks as a colony perished, supporting its use as a colony health indicator. Hive temperature and CO_2_ concentration show diurnal patterns, indicating that CO_2_ can record respiratory activity and daily colony-level organization [73].

Honey preservation and brood development are also linked by gas exchange. While honey cappings are almost gas-impermeable and help prevent fermentation of stored honey, brood cappings have microscopic pores that permit gas exchange for developing brood [88]. VOC profiling is important for hive health and environmental biomonitoring since [89] showed that portable membrane inlet mass spectrometry can identify volatile compounds in hive air, including expected hive-related compounds and contaminants like signals related to pesticides and pollution. Hive atmosphere volatiles can act as chemical markers of external stress by using beehive air VOC profiles to differentiate environmental contamination patterns in urban and rural contexts [89].

Changes in departures, returns, pollen intake, mortality, and aberrant movement can all be indicators of changes in foraging and stress at the hive entrance, where flight counters and computer vision offer a direct behavioral signal. The majority of the observed fluctuation in foraging activity was explained by temperature and solar radiation and measurement of bee egress using local weather variables. This indicates that flight activity may be modeled against seasonal and short-term environmental change [90]. A precision apiculture platform that combined data on hive weight, temperature, humidity, wind, and weather might assist beekeepers in making better decisions and explain variations in honey output [10].

Because it can identify disruptions in daily activities and bee loss, automated flight tracking is especially pertinent for assessing the risk of pesticides. After being exposed to sulfoxaflor, colony-level flight activity and loss rates were measured using real-time bee counters; it was discovered that higher exposure significantly increased bee losses and decreased daily flight activity [91]. Because sublethal effects can manifest through reduced navigation, foraging, and colony function rather than only immediate mortality, homing flights and other behavioral endpoints are crucial contributions to pesticide risk evaluation [92]. By generating continuous daily loss rate data that are challenging to obtain with traditional dead-bee traps, automated bee counters can enhance regulatory assessment [93].

Entrance surveillance goes beyond simple counts thanks to computer vision. In order to enable both colony-level and individual-level surveillance, Rodriguez et al. [94] created an automated video system that uses convolutional neural networks to recognize entries, exits, body parts, pollen carrying, and marked-bee identity. In order to enhance automated interpretation of colony activity at the landing board, [95] employed YOLO-based visual analysis to identify hive entrance behaviors such as foraging, fanning, wash boarding, and defense.

When foraging is altered by predator pressure, entrance-based visual monitoring is also helpful. Invasive *Vespa velutina* predation can increase the risk of winter survival in honeybee colonies by causing homing failure and foraging paralysis [96]. A computer vision can quantify predator–prey interaction at the hive scale by using automated 3D image tracking to demonstrate how hornet density impacts predation success and honeybee flying performance near hive entrances [97].

By connecting respiration, volatile chemistry, foraging intensity, pesticide-related impairment, predator pressure, and seasonal colony dynamics, gas sensing, flight counters, and computer vision work together to extend hive monitoring from internal physiology to outward activities. Table 1 provides a comparison of traditional, molecular, digital, and eDNA-based monitoring techniques.

## 4. Environmental DNA as the Molecular Memory of the Hive

### 4.1. Honey-Derived eDNA

A molecular record of the things the colony has come into contact with, eaten, processed, and stored is provided by honey-derived eDNA. The presence of multi-kingdom DNA from plants, arthropods, fungi, bacteria, and viruses in honey was demonstrated by Bovo et al. [14], indicating that honey can preserve biological signals coming from both colonies and landscapes. Later, Bovo et al. [101] supported honey as a source of environmental and hive-associated signals by using deep shotgun sequencing to identify DNA from 191 creatures, including viruses, bacteria, plants, fungi, protozoans, arthropods, and mammals.

Beyond traditional pollen microscopy, honey DNA can identify plant species for botanical origin and floral landscape reconstruction. Honey samples were differentiated by geographic origin using plant, bacterial, and fungal DNA, with plant DNA providing a substantial distinction across neighboring nations [99]. Honey-borne DNA may recreate the larger ecological context of honeybees, including crop plants, pollinator pathogens, bee-associated microorganisms, and plant pathogens [102]. Although DNA degradation must be taken into account when comparing old and fresh samples, even older honey samples can retain plant, bacterial, and fungal DNA, which indicates that honey can offer temporal insight into previous flower visits and microbial encounters [103].

Additionally, non-invasive disease and parasite surveillance is supported by eDNA generated from honey. In Bulgarian honey samples, Salkova et al. [104] found *Nosema ceranae* DNA, demonstrating the usefulness of honey as a matrix for tracking honeybee diseases. Several infections, including deformed wing virus, chronic bee paralysis virus, black queen cell virus, *Nosema ceranae*, and *Lotmaria passim*, were found in Italian honey sample [105]. Many samples had numerous viruses. In order to determine the botanical content and identify honeybee diseases and parasites, such as *Paenibacillus larvae*, *Nosema ceranae*, *Varroa destructor*, and *Aethina tumida*, Paluoja et al. employed bulk DNA metagenomics of honey [106].

Both the hive microbiome and exposure to the environment are reflected in the bacterial and fungal fraction of honey eDNA. The kind of honey and its physicochemical characteristics shape the bacterial and fungal populations found in raw honey, with the fungal communities exhibiting more variety than the bacterial communities [107]. The molecular profiles might link forage supply with disease risk by connecting honeybee-associated bacteria, fungus, pollen composition, and pathogen susceptibility in anthropogenic environments using 16S rRNA and ITS sequencing [108].

One particularly significant expansion of honey eDNA is the identification of mites and arthropods. *Apis mellifera*, other *Hymenoptera*, *Diptera*, *Coleoptera*, *Lepidoptera*, aphids, and mites were among the arthropods whose DNA was found in honey [14]. Bhasi et al. identified *Varroa destructor*, *Aethina tumida*, *Nosema species*, *Ascosphaera apis*, *Melissococcus plutonius*, and *Paenibacillus larvae* using honey eDNA from Australian samples to screen for arthropod, fungal, and bacterial pests and infections [54]. These investigations collectively demonstrate that honey-derived eDNA can serve as a molecular memory of the floral environment, pathogens, fungi, bacteria, viruses, arthropods, mites, and botanical origin.

### 4.2. Hive Debris eDNA

Because hive debris gathers biological traces from bees, pests, viruses, hive-associated microorganisms, and creatures encountered during foraging, hive debris eDNA is valuable. Henneken et al. [18] repeatedly collected hive debris during pollination and demonstrated that eDNA metabarcoding identified bacterial, fungal, and arthropod communities, including agricultural pests, pathogens, beneficial organisms, biocontrol-associated taxa, hive pests, and organisms important to human health.

Hive debris offers a non-invasive matrix that can identify organisms inside or close to the colony for pest and pathogen surveillance. A total of 4480 amplicon sequence variations from bacteria, fungus, and arthropods were found in debris samples, demonstrating that debris can capture a wide range of biological variety important to hive health [18]. When [109] analyzed eDNA from hive and apiary surfaces, they found DNA from *Aethina tumida*, *Varroa destructor*, and *Melissococcus plutonius* that matched visual indicators of disease and pests in the hives they sampled.

Beyond recognized colony hazards, hive debris eDNA facilitates surveillance. Hive debris can record both colony health signals and landscape-level biological exposure, detecting both beneficial creatures and agricultural pest species not typically associated with hives [18]. In order to support the broader use of bee-collected materials for agricultural biosecurity monitoring, Tremblay et al. demonstrated a related principle using honeybee-collected pollen pellets, where metabarcoding identified plant pathogens and host plants, including *Fusarium*, *Ophiostoma*, *Peronospora*, *Phytophthora*, and *Pythium* [110].

Hive debris and associated hive eDNA techniques are useful for invasive species because they can identify low-abundance organisms without the need for direct visual confirmation. eDNA metabarcoding can aid in the early identification of novel incursions that impact honeybee colonies and their foraging habitat. In order to support its usefulness for biosecurity surveillance, Roberts et al. created a *Varroa destructor* eDNA qPCR assay and showed that eDNA from hive-associated samples could identify Varroa during early hive invasion [100]. All things considered, hive detritus eDNA serves as a small genetic archive of interactions between the hive and the landscape, making it possible to monitor pests, diseases, beneficial creatures, invasive species, and agricultural pests.

### 4.3. Pollen and Wax eDNA

Complementary records of colony exposure are preserved by pollen and wax. While wax is more helpful as a longer-term archive of chemical exposure inside the hive, pollen-derived eDNA directly reflects foraging ecology by identifying the plants visited for pollen and nectar. According to Hawkins et al. [111], DNA metabarcoding can detect a wide variety of plant taxa across numerous families, disclose honeybee foraging preferences, and determine the floral composition of honey. Despite a high plant availability, honeybees only utilized a small portion of the flowering plants in a very diversified landscape, suggesting selective foraging [112]. The honeybee colonies employ similar but distinct plant species for pollen and nectar, with pollen choices exhibiting greater seasonal fluctuation and selectivity [113].

Plant diversity and landscape change can also be described by pollen metabarcoding. Seasonal changes in fodder usage were observed using pollen DNA metabarcoding of honey, with trees being significant in the spring and herbs and shrubs being more prevalent later in the season [114]. Pollen DNA metabarcoding is a high-throughput technique for researching plant–pollinator interactions and ecosystem reactions to global ecological change [115].

The use of bee-collected material as a temporal record of shifting ecological interactions demonstrates that DNA in older honey can disclose past flower visits and microbial encounters [103]. Because residues gathered by foragers reach the colony through pollen loads and bee bread, pollen is also significant for the history of pesticide exposure.

Because residues gathered by foragers reach the colony through pollen loads and bee bread, pollen is also crucial for the history of pesticide exposure. In North American wax and pollen samples, Mullin et al. [116] discovered extensive pesticide residues; wax often contained accumulated miticides and agrochemicals. Bee bread can serve as a seasonal bioindicator of pesticide exposure, with larger residual loading in early-season samples compared to late-season samples [117].

Because lipophilic chemicals can stay in the comb for extended periods of time, wax offers a persistent archive. Every beeswax sample from controlled colonies in New York State had numerous residues of pesticide contamination [118]. According to Hisamoto et al. [119], pesticide residues in honey and beeswax varied according to the land use in the area, with higher exposure in agricultural and urban areas and lower exposure in forests. Within the same hive-based monitoring paradigm, pollen eDNA and wax chemistry connect plant variety, foraging ecology, landscape change, and pesticide exposure history.

## 5. AI and Machine Learning for Colony State Prediction

The analytical layer that transforms hive sensor feeds into colony state forecasts is increasingly AI- and machine learning-driven. Precision beekeeping is a system where IoT sensors gather hive data and analytical models link that data to biological hive states [55]. In contrast to isolated sensor data, combining weight, sound, temperature, humidity, CO_2_, and weather measures can offer a more comprehensive understanding of colony status [56].

One of the most advanced AI applications for hive monitoring is acoustic classification. Zgank et al. [120] distinguished between normal and swarming hive conditions using machine learning models and auditory features for Internet of Things-based monitoring. In order to identify swarming events from sound, Dimitrios et al. [121] compared convolutional neural networks, support vector machines, and k-nearest neighbors. CNN models performed particularly well on MFCC-based inputs, demonstrating good swarm-prediction performance utilizing acoustic characteristics [122].

AI that uses sound can also identify anomalies relating to queens. Non-invasive notifications for a condition typically necessitates inspection by identifying queenless colonies using hive acoustics and machine learning algorithms [123]. Książek et al. [124] demonstrated how model interpretation can contribute to the transparency of acoustic monitoring by applying explainable machine learning to hive sound and identifying key spectral bands for diurnal activity detection.

AI monitoring is extended to observable hive activity and structural issues through image recognition. Sledevič et al. [95] achieved great detection performance at real-time speed by using YOLOv8-based computer vision to identify entrance behaviors like foraging, fanning, wash boarding, and defense. Using picture collection and embedded or cloud-based processing, ref. [125] developed a CNN-based smart monitoring system for bee swarming detection. Ref. [126] identified hive health anomalies, such as *Varroa destructor*, hive beetles, ant issues, and absent queen status, using a Mobile Net-based deep learning model.

Since many beekeepers are unable to implement costly cloud-based technologies, low-cost AI is crucial. A good classification performance may be attained with little computational cost by comparing machine learning models for acoustic colony state classification on constrained hardware [127]. In order to facilitate beehive management on low-power devices, ref. [128] introduced an IoT edge-learning system that uses hive sounds and TinyML-style processing. While pointing out ongoing limitations in data availability, generalization, and deployment, Sucipto et al. reviewed TinyML applications in beekeeping and identified monitoring hive conditions, identifying bee behavior, detecting pests and diseases, and forecasting swarming as important low-power AI directions [129].

AI-based dashboards, explainable models, anomaly detection, and shared learning across apiaries are still in the early stages of development at the system level. In addition to highlighting unresolved issues with integration, dataset standardization, and large-scale implementation, Šabić et al. [13] discovered significant growth in deep learning, computer vision, and multimodal sensor fusion for smart beehives. Precision beekeeping systems offer a lot of potential, there are a number of obstacles to overcome, including cost, operator readiness, and the possibility of drawing false conclusions when just a portion of the hives are monitored [16]. AI is, therefore, currently capable of classifying sound, pictures, and sensor readings; however, more robust data fusion, explainability, cross-regional validation, and cost-effective deployment are needed for trustworthy early warning displays.

## 6. Exposomics: Chemical Fingerprints of Colony and Ecosystem Risk

The chemical risk layer required to interpret eDNA and hive sensors combined is added by exposomics. Pesticides, herbicides, fungicides, acaricides, veterinary medications, heavy metals, microplastics, PFAS, air pollutants, plant toxins, plasticizers, and mixed residues are not considered isolated contaminants in this framework; instead, they are interpreted as exposure fingerprints that may help explain abnormal flight activity, impaired thermoregulation, decreased weight gain, altered brood performance, pathogen shifts, and residues building up in wax, pollen, bee bread, or honey. Among the most well-documented chemical stressors in honeybee systems are pesticides. In North American wax, pollen, bee, and hive samples, 121 pesticides and metabolites were detected; wax and pollen frequently revealed multi-residue contamination; this study is, nonetheless, significant because it demonstrated that bees are not often exposed to single compounds in actual apiaries [116].

McArt et al. [130] demonstrated that pesticides and non-focal pollen sources, rather than the crop being pollinated, frequently drove pesticide risk in bee bread during apple pollination, connecting exposure risk to the larger foraging landscape. Pollen can recreate short-term exposure pulses that may otherwise be missed by sporadic sampling, as demonstrated by [131] who discovered 73 pesticides in daily pollen loads across agricultural sites. In a similar vein, serial exposure maxima throughout the season and repeated pesticide detections in daily pollen samples were observed [132].

Because they frequently coexist with insecticides in actual exposure mixes, herbicides and fungicides are also significant. AMPA and glyphosate-based herbicide residues were detected in bee bread and wax; glyphosate was found more frequently in bee bread than in wax, demonstrating that hive matrices can be used to track herbicide exposure [133]. Greek honey, pollen, and bee bread were found to contain pesticide and metabolite residues, such as coumaphos, imidacloprid, acetamiprid, amitraz metabolites, tau-fluvalinate, and pyrethroids; pollen and bee bread accumulated more active chemicals than honey [134]. Bee bread is a seasonal bioindicator of pesticide exposure; bee bread in Slovakia had larger residue loading early in the season than later, with fungicides dominating many samples [117]. Because lipophilic substances can linger in comb, wax is an important matrix for exposure history. El Agrebi et al. [135] discovered 54 pesticide and veterinary medication residues in Belgian beeswax and noted that these residues were frequently lipophilic chemicals or in-hive products; their risk model associated certain wax contaminants with the likelihood of bee mortality.

Wilmart et al. [136] created exposure scenarios for residues in beeswax, demonstrating how cell contact, contaminated larval feeding, or wax chewing might expose larvae and adults to contaminated wax. Beeswax had higher levels of acaricide contamination than honey, brood, or bees, demonstrating that wax can hold onto treatment residues during varroosis management [137]. All beeswax samples from managed colonies in New York State included pesticide residues, with commercial operations exhibiting the highest residual counts [138].

Because disease and Varroa control can introduce antibiotics and acaricides into honey and wax, veterinary residues are a part of the hive exposome. According to Eissa et al. [139], the majority of EU RASFF honey notifications from 2002 to 2022 were unapproved veterinary pharmaceutical residues, including chloramphenicol, streptomycin, sulfathiazole, tylosin, and sulfadimidine.

*Paenibacillus larvae* is still a serious colony disease; the use of antibiotics against American foulbrood raises issues about residue and resistance [140].

Heavy metals offer an additional indicator of exposure from urban, industrial, and soil sources. During wax foundation processing, it was demonstrated how metals, such as arsenic, cadmium, lead, and mercury, in beeswax can be redistributed [141]. After reviewing environmental contaminants in honey and bee products, The most concerning contaminant groups are pesticides and hazardous metals, particularly when samples come from urban or industrialized areas [142]. Heavy metals, pesticides, antibiotics, and acaricides are significant pollution-related concerns for honeybees and bee products [143].

The exposome is expanded beyond traditional agrochemicals by microplastics and PFAS. In their review of microplastics in honeybee systems, air, water, soil, pollen, hive plastics, and beekeeping materials were identified as potential exposure routes, and possible effects on mortality, sucrose response, olfactory learning, memory recall, colony performance, gut microbiota, and viral infection were reported [144]. Perfluoroalkyl compounds were found in European honey samples. The majority of the samples included perfluoroalkyl carboxylic acids, with larger amounts in samples from industrial areas [145]. Pesticides, persistent organic pollutants, polycyclic aromatic hydrocarbons, medicines, and microplastics were among the contaminant cocktails in honey. They emphasized the need for analytical techniques that can identify several contaminant classes at once [146].

Because foragers can carry pollution-related stress back to the colony through the atmosphere, air pollutants are important. Air pollution may impact both individual bees and colony-level performance, according to [29], who subjected colonies to diesel exhaust and observed alterations in neuronal health markers in foragers and subsequent colony weight loss compared with controls. Ozone exposure connects atmospheric pollution with sensory and metabolic stress: ozone exposure can change antennal responses to floral volatile organic compounds (VOCs), decrease olfactory recall, and impact antioxidant responses in honeybees [147].

Because they enter bee products from floral sources, plant poisons and naturally derived hazardous chemicals are included in the hive exposome. Plant-derived toxins such as pyrrolizidine alkaloids, tropane alkaloids, matrine alkaloids, grayanotoxins, gelsemium alkaloids, and tutin were identified as potential natural hazards after reviewing understudied food safety risks in bee products [148]. Honey may include bioactive substances with toxicological significance, such as pesticide residues, plant toxins, trace metals, and degradation products [149]. Connecting chemical fingerprints with hive function—rather than just detecting residues—is exposomics’ greatest systems biology utility. Abnormal colony signals may need to be evaluated at the landscape scale; pesticide risk during blueberry pollination was frequently linked to off-farm exposures and landscape composition instead of just the target crop [150].

Pesticide residues in honey and beeswax varied according to land use, with woods associated with lower exposure and agricultural and urban landscapes associated with higher exposure [119]. The assumption that sensor records and chemical profiles should be evaluated jointly is supported by [151], who demonstrated that colonies transferred through various landscapes differed in brood, adult bees, hive temperature, weight gain, and pesticide residues in hive matrices.

Exposomics is crucial for interpreting eDNA and pathogen surveillance since chemical exposure can interact with infections and microbial changes. Plant-use patterns and microbial signatures can change with environmental conditions by connecting fodder availability, bacterial and fungal profiles, and disease susceptibility in honeybees [108]. The different stressor syndromes were predicted by pollen-foraging behavior: narrower foraging patterns were linked to higher xenobiotic exposure, while diversified plant foraging was linked to higher pathogen exposure [152]. Pasture identification and chemical exposure may be assessed in the same field-realistic monitoring scheme by combining eDNA analysis of bee pollen plant sources with pesticide residue analysis in honey and pollen [153].

Therefore, residues should be considered as explanatory factors for sensor anomalies in a practical early warning model. When combined with contaminated pollen or bee bread, abnormal flying activity may be a sign of exposure to pesticides or air pollution. Reduced hive weight gain could be the result of resource pollution at the landscape level or poor feeding. Reduced worker force, brood stress, or physiological disturbance caused by contaminants could all be indicated by altered thermoregulation. Colonies under chemical stress may be distinguished from colonies under predominantly infectious pressure by increased pathogen DNA or changed microbial signatures in honey, pollen, or debris. Because toxicants can impact workers, brood, honey, bee bread, and structural wax while interacting with nutrition, infectious disease, and management factors, toxicant interpretation in apiaries must take colony-level medicine into consideration [138]. The colony serves as both the exposed organism and the ecosystem risk sampler in this systems architecture. Table 2 summarizes the four phenotypic layers of the H-BEWS paradigm.

## 7. Building the Systems Biology Framework

### 7.1. Digital Phenotype

The digital phenotype, a continuous, non-invasive, real-time depiction of colony physiology and behavior obtained from sensor streams and imagery, is the initial layer of a systems biology framework for honeybee-based early warning. Digital phenotyping records the temporal dynamics of colony function and stress responses at high resolution, in contrast to routine hand examinations. Because they represent metabolism, brood rearing, foraging effort, colony cohesiveness, and environmental interactions prior to the onset of symptoms, these signals serve as functional biomarkers in precision apiculture [12]. Therefore, the digital phenotype offers the first interpretable layer in an integrated framework that connects genetic, chemical, and ecological risk to physiological status.

Because it captures nectar influx, food consumption, foraging activity, swarming, robbing, and colony decline, hive weight is one of the most useful and popular digital phenotypes. Within-day and seasonal colony activity patterns related to nectar flow and foraging can be identified using continuous hive weight monitoring [154]. While chronic weight loss may suggest food scarcity or colony weakening, sharp short-term losses may indicate swarming or robbery [12]. Weight increase trajectories have been used to assess the availability of fodder at the landscape level among apiaries and to estimate colony productivity [151]. As a reliable indication of colony phenology and ecosystem resource pulses, next-generation colony weight monitoring has also been suggested [73]. Weight data in conjunction with meteorological factors enhances the assessment of variations in honey production and management choices in precision platforms [10].

Because brood-rearing colonies actively control the temperature of their brood nests within a specific range, usually between 34 and 36°C, temperature is a key physiological indication. Broodlessness, colony weakening, queenlessness, or approaching collapse can all be indicated by deviations from this range. Colony stress and decreased thermoregulatory capacity are frequently reflected in temperature variability rather than absolute temperature [12]. Internal hive temperature patterns have been utilized to categorize colony conditions, identify brood-rearing times, and identify broodless states [155]. Resilience to external temperature extremes can provide early warning signs of instability and collapse, according to recent analysis of temperature time series [67]. By enabling non-invasive monitoring of brood dispersion, colony clumping, and heat anomalies linked to swarming or disease, thermal imaging expands on this trait [156].

Complementary information on nectar dehydration, ventilation, brood development, and disease-favorable circumstances is provided by humidity. Workers actively control the relative humidity inside the brood nest, which is crucial for the development of the larvae and the successful emergence of the brood. While unusually low humidity can hinder brood survival, elevated humidity may be a sign of inadequate ventilation, nectar ripening activity, or external moisture incursion [157]. Since evaporative dehydration is necessary for honey ripening, variations in humidity can potentially be a sign of changes in nectar processing [12]. Humidity can be used to differentiate between active nectar curing, environmental stress, and colony malfunction when combined with temperature and weight.

Vibrational and acoustic characteristics shed light on collective behavior and colony communication. The distinctive sound and vibration patterns produced by honeybee colonies are linked to worker activity, queen piping, waggle dances, brood cycles, and swarming preparation. Queenlessness, swarming, and colony disruptions have all been identified using acoustic categorization [158]. Colony mobility patterns and brood cycle periodicity can be discovered by vibrational analysis [83]. Machine learning techniques are capable of automatically identifying pre-swarming signs such as queen piping and worker-produced “tooting” or “quacking” vibrations [84]. Acoustic signals associated with disturbances could be signs of environmental stressors, beekeeper manipulation, or predator assaults. Sound and vibration give an early signal of behavioral changes at the colony level before changes in production become apparent because they record social interactions.

Foraging intensity, orientation behavior, and colony workforce performance are all directly correlated with flight activity at the hive entrance. Pollen loads, anomalous flight routes, and incoming and exiting bees can all be measured using optical counters, RFID systems, and computer vision technologies. Reduced outgoing flights could be a sign of illness, pesticide exposure, food scarcity, or weather restrictions. Pesticide-related navigation impairment has been associated with higher rates of unsuccessful returns or confused flight patterns [159]. Hornet attacks and other predator pressure can change defensive behavior at entry and decrease regular traffic [151]. The availability of floral resources and colony phenology are also reflected in seasonal variations in foraging intensity. Thus, an exterior behavioral phenotype closely associated with internal colony status is provided by continuous flight tracking.

Scalar sensors are unable to capture the spatial and structural information that image-based phenotyping adds. From interior or entrance photos, computer vision can determine the number of adult bees, brood area, comb occupancy, pollen reserves, and queen presence [94]. Abnormal brood patterns, obvious illness symptoms, wax moth damage, and hive incursions can all be found using thermal and RGB imaging. In order to detect Varroa on bees and comb surfaces, automated image recognition methods have also been created [60]. Image-based systems can identify invasive predators, detect drifting behavior, and categorize pollen-carrying bees at the hive entrance. Thus, image streams add morphological and behavioral characteristics to the digital phenotype.

When these streams are combined, digital phenotyping becomes most valuable. Reduced flying activity and weight loss could be signs of pesticide stress or forager mortality. Rather than colony failure, increased humidity with stable or increasing weight may indicate nectar cure. Reduced acoustic activity and temperature instability could be signs of colony collapse or brood loss. Abrupt weight declines, changes in internal temperature gradients, and increases in piping signals can all be used to identify pre-swarming situations. By lowering false alarms and enhancing biological interpretation, sensor fusion increases early warning systems’ sensitivity and specificity [160]. Longitudinal modeling of colony resilience is also made possible by digital phenotyping. Continuous multisensor datasets have connected phenotypic characteristics such brood area, population size, Varroa infection, sanitary behavior, and winter mortality to audio, temperature, and humidity variables [77]. These datasets enable machine learning algorithms to predict risk trajectories and infer latent colony states. As a result, the digital phenotype serves as the systems biology framework’s functional front end, recording dynamic colony responses in real time and serving as the initial signal that can initiate molecular sampling, exposomic analysis, or intervention.

The hive is transformed by this digital layer into a constantly observed living organism whose physiological and behavioral outputs may be measured reliably and on a large scale.

### 7.2. Molecular Phenotype

The second layer is the molecular phenotype, which is the biological identity of the colony and its surroundings as determined by eDNA, metagenomics, pathogen DNA/RNA, floral DNA, and pest DNA. Molecular signals help explain the biological agents the colony has come into contact with, while sensors describe how the colony behaves.

A kingdom-wide record of colony exposure is provided by honey and eDNA produced from hives. Signals from bacteria, fungus, arthropods, honeybee-associated organisms, mites, plant sources, and viral sequences can be found in honey DNA [14]. Bovo et al. demonstrated that honey may contain both hive and environmental biological signatures by using shotgun sequencing to identify DNA from 191 creatures, including viruses, bacteria, plants, fungi, protozoans, arthropods, and mammals [101].

This layer is expanded by metagenomics from focused detection to comprehensive biological profiling. In order to determine the botanical composition and identify honeybee diseases and parasites, such as *Paenibacillus larvae*, *Nosema ceranae*, *Varroa destructor*, and *Aethina tumida*, Paluoja et al. [106] employed honey bulk DNA metagenomics. Hive debris eDNA metabarcoding may identify bacterial, fungal, and arthropod populations, including pathogens, pests, beneficial species, and taxa that are significant to agriculture [18].

The molecular phenotype’s diagnostic core is made up of pathogen DNA and RNA. DNA and RNA together can enhance the resolution of pathogen and exposure signals by using combined eDNA and eRNA profiling of royal jelly to identify bacterial pathogens, mites, pests, and numerous bee viruses [53]. The usefulness of honey eDNA for surveillance of less frequently observed bee diseases can be justified by proving that it can identify *Spiroplasma apis* and *Spiroplasma melliferum* [54].

Colony biology and landscape ecology are linked by floral DNA. According to Hawkins et al. [111], DNA metabarcoding can indicate foraging preferences and determine the floral composition of honey. Pathiraja et al. [51] traced the floral and geographical origins of honey using eDNA metabarcoding of bacterial, floral, and entomological DNA. Honey’s DNA can characterize variations in flower visits and microbial interactions over time, making floral DNA valuable for monitoring changes in the landscape and the history of ecological interactions [103].

The early-warning element of this layer is pest DNA. In order to demonstrate that honey may identify harmful hive pests, ref. [161] created honey eDNA assays for *Aethina tumida* and *Galleria mellonella*. eDNA swab sampling can detect even a single tiny hive beetle in a beehive colony following brief exposure, demonstrating the sensitivity of eDNA as a biosecurity tool [162]. In order to support molecular surveillance of mites, Roberts et al. showed that *Varroa destructor* eDNA may be found from hive-associated samples, especially during early invasion [100].

Digital abnormalities are given biological significance by this molecular phenotype. In order to differentiate between colony disease and low feed, reduced flight activity can be evaluated in conjunction with floral DNA. To determine whether brood stress is linked to infection, temperature instability can be combined with pathogen DNA/RNA. Varroa, small hive beetle, or wax moth pressure can be identified by analyzing weight loss using pest DNA. The molecular phenotype therefore transforms sensor-based warning into biological diagnosis.

### 7.3. Chemical and Ecological Phenotypes

The contaminant fingerprint that the colony and its products carry is represented by the chemical phenotype. By detecting residues in honey, wax, pollen, bee bread, bees, and hive air, it establishes a connection between hive performance and the chemical environment. Wax and pollen can contain complex mixes of miticides, insecticides, fungicides, and herbicides, which shows that colony exposure is typically multi-contaminant rather than single-compound [116]. Beeswax can hold onto veterinary medication and pesticide residues, which makes wax a crucial long-term matrix for analyzing colony exposure history [133].

Currently, the most compelling evidence for this layer comes from pesticides, fungicides, and herbicides. Recurring pesticide peaks in bee pollen during the active season and daily pollen loads can capture pesticide exposure pulses across agricultural landscapes [131,132]. Herbicide monitoring as part of the hive chemical phenotype is supported by [135], who discovered glyphosate and AMPA in bee bread and wax. Seasonal variations in bee bread residue loads were demonstrated by [117], with early-season samples exhibiting higher pesticide burdens than late-season samples. Beyond agricultural residues, the chemical phenotype is expanded by metals, PFAS, plastics, and volatile organic compounds. Wax can collect inorganic pollutants, discovery of arsenic, cadmium, lead, and mercury in beeswax [141]. PFAS were found in European honey samples by [145], with higher quantities seen in samples from industrial areas. The routes of microplastic exposure in honeybee systems document possible impacts on colony performance, learning, memory, gut microbiome, and virus infection [144]. Hive air VOC profiling may identify environmental contamination signals and volatile substances, turning the hive atmosphere into a chemical matrix for real-time risk assessment [30].

The colony is positioned within its landscape environment by the ecological phenotype. What bees gather, what pollutants they come across, and how resilient the colony can be are all influenced by floral diversity, land use, climate, crop calendars, and fragmentation. Even in a very diversified landscape, honeybees only utilized a small portion of accessible flowering plants, suggesting that floral availability and actual forage use are not the same [112]. Jones et al. [114] linked colony nutrition to seasonal floral calendars by demonstrating that fodder utilization varies with the season, with trees being more significant in the spring and herbs and shrubs becoming more significant later. Honeybees choose distinct plant genera for pollen and nectar, demonstrating the need for both resource type and season in ecological interpretation [113].

Because exposure and nutrition are landscape-level processes, land use and fragmentation are crucial. In contrast to the target crop alone, off-farm exposure was the primary factor driving pesticide risk during blueberry pollination [150]. The nearby land-use categories influenced pesticide residues in honey and beeswax, with woods having lower exposure and urban and agricultural regions having higher exposure [119]. In order to facilitate the integration of landscape data with hive-level phenotypes, Meikle et al. [151] connected landscape context with brood, adult bee mass, hive temperature, weight gain, and pesticide residues.

The ecological layer is completed by climate and crop calendars, which provide flowering times, windows for the application of pesticides, forage gaps, and conditions that are conducive to disease. Honeybee foraging activity is significantly influenced by local weather factors, particularly temperature and sun radiation [90]. Global change can modify plant–pollinator–pathogen networks, underscoring the significance of connecting ecological change with pathogen surveillance [163]. Pests, viruses, pesticide mixtures, habitat loss, urbanization, climate change, and beekeeping movement are some of the interrelated stressors that affect honeybee health [164].

The ecological and chemical phenotypes work together to explain why various landscapes may have different reasons for the same digital abnormality. Reduced weight increase could be the result of a crop calendar gap, drought, pesticide exposure, or insufficient feed. Unusual flying activity could be a sign of predator pressure, pesticide exposure, or weather stress. Chemical combinations, habitat fragmentation, and nutritional stress can all increase pathogen changes. These layers transform the hive from a monitored box into a biological sensor that is connected to the landscape.

### 7.4. Predictive Decision Layer

Hive signals are translated into action via the predictive decision layer. This layer combines digital, molecular, chemical, and ecological data to create risk scores that can categorize colonies as intervention-ready, watch-listed, or stable. In order to forecast internal hive conditions and support alarms for uncommon colony states, Robustillo et al. [25] demonstrated how predictive models utilizing hive temperature, humidity, weight, and weather data can be used. Because of this, monitoring turns into early warning at the decision layer.

One variable should not be the only basis for an early warning threshold. When combined with decreased flying activity, unstable temperature, pathogen DNA, or pesticide residues, a weight loss during bad weather may become more significant.

The B-GOOD project is a model for automated predictive health monitoring that creates a Health Status Index by integrating colony data, environmental data, disease burden, brood, honey harvest, floral resources, and machine learning [165]. In order to incorporate several aspects of colony health, such as queen performance, demographics, in-hive products, disease, infection, infestation, management, and environmental causes, ref. [5] also stressed the need for a Health Status Index.

Only after the system determines the most likely risk pathway should management advice be produced. Treatment planning and diagnostic confirmation may be advised if the primary signal is increasing Varroa or pathogen DNA. Relocation or additional feeding may be advised if decreased weight gain with low floral DNA diversity is observed. It might be advisable to look into exposure and get in touch with local growers if pesticide residues are present together with unusual flying activity. Beekeeping management techniques, including as chemical control, comb replacement, supplemental feeding, worker number adjustment, and beekeeper experience, can affect colony status and ought to be incorporated into risk management models [166].

When numerous apiaries exhibit the same risk pattern throughout an area, policy warnings become feasible. According to Rortais et al. [167], bee risk assessment has to shift toward a comprehensive evaluation of several stressors, such as chemical combinations, biological risks, management, and landscape context. B-GOOD is a coordinated data platform that supports adaptive management, beekeeper guidance, and EU-wide bee health evaluation [168]. These devices could notify authorities of disease transmission, forage failure, invasive pest emergence, and chemical exposure in the area.

The framework’s most comprehensive output is the ecosystem health index. A landscape-level interpretation should incorporate colony performance, floral variety, pathogen pressure, contaminant burden, pest DNA, and land-use context. Smart beehive technologies are progressing toward multimodal sensor fusion and predictive intervention, according to [13]. However, they also pointed out that there are still issues with large-scale implementation, integration, and dataset standardization. In a similar vein, in order to prevent drawing false findings from incomplete monitoring, precision beekeeping systems need to be economically beneficial, scalable, and properly evaluated [16]. Therefore, by converting biological complexity into graded alerts, management alternatives, policy signals, and ecosystem health assessment, the decision layer completes the systems biology framework.

## 8. H-BEWS Framework: From Colony Health to Ecosystem Surveillance Applications, Standardization, and Societal Dimensions

The Honeybee-Based Early Warning System (H-BEWS) represents a novel integrative framework that unifies multiple monitoring layer digital sensors, molecular eDNA, chemical analysis, and ecological data into a predictive, early warning system for colony and ecosystem health. Unlike previous monitoring approaches, which often focus on a single technology or are restricted to reactive hive assessments, H-BEWS provides multi-layer integration, enabling sensor anomalies to trigger targeted molecular or chemical analyses and link environmental context to colony responses. Prior frameworks, such as traditional hive inspections [12], smart hive technologies [13], and IoT-based sensor networks [17,28], primarily monitor either colony health or environmental factors in isolation. By contrast, H-BEWS combines these approaches, enhancing predictive capability and providing actionable insights within a One Health paradigm, where pollinator, ecosystem, and human health are interconnected. It is possible to think of honeybee colonies as dispersed biological sentinels that produce quantifiable warning signals based on colony activity, hive material, forage inputs, diseases, and pollutants. Smart hive sensors, eDNA sampling, exposome profiling, AI-based risk prediction, beekeeper dashboards, and regional surveillance networks would all be integrated into an operational model by a Honeybee-Based Early Warning System, or H-BEWS. While hive debris, honey, pollen, wax, and air samples can provide molecular evidence of plants, microbes, arthropods, pests, and pathogens, sensor streams like hive weight, temperature, humidity, sound, gases, flight activity, and weather can describe the digital phenotype of the colony [9,18,19]. In this concept, aberrant sensor patterns would be used as triggers for specific molecular and chemical tests rather than as definitive diagnoses. This would enable the evaluation of pathogen DNA/RNA, floral resource shifts, mite indicators, pesticide residues, and landscape conditions in addition to a colony exhibiting restricted flight, disrupted thermoregulation, poor weight increase, or aberrant auditory activity.

In situations where single observations are insufficient, H-BEWS’s colony health value is strongest. Stress related to varroa, virus outbreaks, queenlessness, swarming, nutritional stress, brood failure, weak-colony formation, aberrant overwintering, and collapse risk are frequently the result of multiple interrelated factors rather than a single obvious cause. Particularly through deformed wing virus and associated viral complexes, *Varroa destructor* is frequently associated with viral amplification and colony loss [98,169,170]. Therefore, eDNA and pathogen testing could clarify whether the signal is linked to mites, viruses, bacteria, fungi, or nutritional disruption, while continuous monitoring could identify colony-level alterations before symptoms become apparent. The use of sensor data as an early-warning layer rather than a passive record is supported by real-time bee counters, which have already demonstrated that flight activity and bee loss rates can disclose pesticide-related risk at the colony level [91].

The same method can be used for ecological surveillance in addition to colony management. Because honeybees graze in both agricultural and semi-natural environments, signs of floral diversity, crop blossoming, invading creatures, agricultural pests, microbial populations, and environmental disturbance may be found in their products and hive wastes. While hive debris metabarcoding can identify arthropod, fungal, and bacterial communities important to hive health and the broader agroecosystem, honey-based biosurveys demonstrate that honey can preserve ecological information about botanical origin and biological interactions [18,19]. It has also demonstrated how bee activity, floral resource variety, and chemical exposure may be evaluated jointly during crop pollination using pollen metabarcoding in conjunction with video-based activity monitoring and pesticide residue testing [171]. Because of this, H-BEWS is useful for monitoring biodiversity, identifying invasive species, monitoring agricultural pests, mapping pollution, evaluating land use, detecting climate-related floral mismatch, and monitoring One Health.

Standardization is crucial to the dependability of such a system. Before data from various apiaries can be compared, it is necessary to unify sensor calibration, sampling frequency, metadata structure, seasonal interpretation, colony genetics, apiary management, regional climate, contamination control, bioinformatics pipelines, and AI model benchmarking. According to Health et al. [5], the HEALTHY-B framework highlighted that a single assessment is insufficient to assess colony health; instead, several characteristics are needed, such as queen status, demography, hive products, infection, infestation, management, and environmental causes. Reviews of precision beekeeping reveal that while sensor platforms are promising, they are nevertheless constrained by factors including cost, operator training, insufficient field validation, and inconsistent access to commercial features like flight monitoring [16]. AI predictions may stay isolated, non-transferable, and challenging to understand in the absence of shareable datasets and repeatable analytical processes.

From the start, H-BEWS should incorporate ethical, social, and economic considerations. Whether such systems can be implemented responsibly depends on a number of factors, including beekeeper data ownership, privacy of apiary locations, fair access for small-scale beekeepers, open-source sensor options, farmer–beekeeper data sharing, and regulatory use of bee-derived environmental information. Smart hive technologies have the potential to enhance pollination management, but if data systems are managed by external platforms or primarily intended for large commercial operations, they may potentially place financial and labor strain on beekeepers [172]. Therefore, a reliable early warning system should combine scientific accuracy with affordable access, transparent algorithms, safe data administration, and explicit guidelines for the application of environmental and colony alarms.

For beekeepers, researchers, and environmental decision-makers, H-BEWS provides a useful path from observation to prediction: the hive becomes a living sampler, sensors identify physiological change, molecular and chemical tools identify likely drivers, and AI transforms combined evidence into actionable risk warnings.

## 9. Future Research Agenda

Future research should focus on fully operationalizing H-BEWS as a predictive, multi-layer early warning system. Key priorities include standardizing sensor calibration, metadata collection, and bioinformatics pipelines to ensure interoperability across devices and laboratories [17]. Low-cost and community-based monitoring approaches should be explored to allow small-scale beekeepers to participate in H-BEWS networks, extending predictive monitoring to broader geographic areas [28]. Ensuring interoperability and standardization of data streams across sensors, labs, and community networks is essential to allow small-scale beekeepers to participate effectively in H-BEWS, while maintaining predictive accuracy and comparability of data. Integrating digital, molecular, chemical, and ecological data will enhance the system’s ability to anticipate colony stress and environmental hazards, supporting proactive interventions and improving ecosystem and human health outcomes [20,21,22]. Future research should transition H-BEWS from discrete monitoring instruments to a field-ready, validated surveillance strategy. Because honey, hive debris, pollen, and bee tissues can contain multi-kingdom DNA signatures from plants, microbes, viruses, parasites, and pests, making them suitable for non-invasive colony and landscape surveillance, real-time molecular monitoring is a critical priority [14,101,106]. Future research should define temporal activation thresholds, whereby anomalies detected by digital sensors trigger targeted molecular or chemical sampling. Synchronizing sensor alerts with eDNA and chemical measurements will enhance the predictive utility of H-BEWS, enabling earlier detection of colony stress or environmental hazards. Because third-generation sequencing has previously shown seasonal viral and bacterial infection patterns in honeybee hemolymph, including pathogens not included in regular screening, nanopore sequencing should be examined directly in apiaries [165].

Multimodal AI models that integrate sensor signals, pictures, acoustics, molecular profiles, chemical residues, weather, and landscape data should also be developed in the following phase. Hive weight, temperature, humidity, sound, gasses, flight activity, and external environmental factors are already used by current precision beekeeping systems; however, integration across data types is still limited [9,55]. Colony state forecasting is possible according to predictive models of internal hive conditions and swarming, but these models require more extensive validation across climates, bee genotypes, management systems, and disease pressures [25,84].

In order to replicate colony growth, thermoregulation, foraging, pathogen dissemination, pesticide exposure, and overwintering danger, digital twins of honeybee colonies should be created. The potential for more mechanistic colony models is supported by robotic honeycomb investigations that demonstrate that continuous internal sensing and closed-loop thermal interaction can capture colony-level thermal organization [173,174]. Because colony losses are frequently influenced by the combined impacts of mites, viruses, pesticides, nutrition, climate, and management rather than by a single stressor alone, these models ought to be connected with exposome–pathogen interaction investigations [23,170].

For the system to be helpful beyond individual apiaries, international surveillance networks are required. Synchronized sensor streams, pathogen loads, eDNA profiles, chemical residues, weather, floral resources, land use, treatment histories, and colony outcomes should all be included in standard databases. Queen performance, colony demographics, hive products, infection, infestation, management, and environmental factors are currently highlighted by harmonized health assessment methods; however, these metrics must be linked to real-time digital and molecular data [5]. Future research should establish decision thresholds that inform beekeepers when a signal calls for laboratory confirmation, treatment, feeding, queen replacement, removal, or inspection. Policy choices about pesticide exposure, invasive pests, biodiversity change, and agricultural landscape risk may also benefit from bee-derived environmental data, but only after validation, with open governance, and explicit guidelines for data ownership and use.

**Figure 3:**
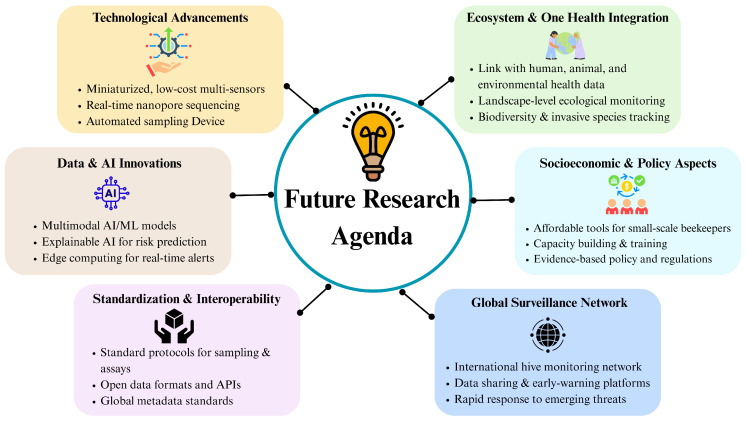
Future research agenda for the Honeybee-Based Early Warning System (H-BEWS). Key priorities include standardizing sensors and metadata, integrating digital, molecular, chemical, and ecological data, developing multimodal AI models, defining activation thresholds for molecular/chemical sampling, creating digital twins of colonies, and enabling participation of small-scale beekeepers through community monitoring networks. This figure highlights actionable goals to advance predictive, early-warning monitoring within a One Health framework.

Instead of only explaining colony failure after it happens, the primary objective is developing a system that anticipates danger early enough for action. Advances in real-time nanopore sequencing, multimodal AI models, and the creation of digital twins of colonies represent promising avenues to strengthen predictive accuracy within H-BEWS [28]. Coupled with global surveillance networks, these tools can provide early warning insights for both colony wellbeing and environmental monitoring [17]. Emphasizing the One Health perspective, H-BEWS should not only detect stressors affecting honeybees but also provide actionable information relevant to biodiversity, agriculture, and human health, ensuring that interventions benefit both pollinators and the ecosystems on which they depend [20,21]. The future research agenda for H-BEWS will highlight key priorities including sensor standardization, multimodal data integration, AI model development, and community-based monitoring for small-scale beekeepers, as shown in Figure 3.

## 10. Conclusions

No single method fully reflects the complexity of colony and ecosystem health, as this review has shown by tracking the development of honeybee monitoring from traditional visual inspection through the molecular and digital ages. Conventional approaches rely on observers and identify issues too late. Although they do not produce results in real time, molecular techniques allow for early infection detection. Although they offer continuous data, digital sensors have difficulties with cost and calibration.

Four phenotypic levels are included in the proposed Honeybee-Based Early Warning System (H-BEWS). Real-time signs of swarming, thermoregulation, and colony activity are provided by the digital phenotype. Pathogens, pests, and floral resources are identified by the molecular phenotype, which is derived from eDNA analysis of honey, debris, pollen, and wax. In hive matrices, the chemical phenotype finds heavy metals, acaricides, and pesticides. The colony’s ecological phenotype situates it within the landscape context of crop calendars, weather, and land use. Risk classification is made possible when molecular and chemical testing is triggered by sensor anomalies, and management suggestions are derived from the most likely stressor pathway.

There are still a number of difficulties. Regional standardization is necessary for bioinformatics pipelines, AI models, and sensor calibration. Access is restricted for small-scale beekeepers due to cost. Transparent governance is necessary for data ownership and the regulatory use of information obtained from bees. Future priorities will include digital twin simulations of colony dynamics, multimodal AI models combining sensor and molecular data, real-time nanopore sequencing in apiaries, and global surveillance networks with standardized databases.

In conclusion, exposomics, artificial intelligence, smart sensors, and environmental DNA have all advanced to a point where integration is now practically required. The honeybee colony can function as a live sensor, molecular recorder, and chemical sampler over the landscape in addition to being a pollinator. H-BEWS can turn honeybees into an early warning system for colony health and ecosystem monitoring through interdisciplinary cooperation.

## Figures and Tables

**Table 1:** Comparison of honeybee colony monitoring modalities.

Modality	Temporal Resolution	Invasiveness	Key Parameters	Limitations	References
Classical (visual)	Sporadic (days–weeks)	High	Brood area, queen status, food reserves, pest counts	Observer- dependent, late detection	[4,37]
Molecular (PCR/qPCR)	Episodic	Moderate	Viral RNA, bacterial DNA, Nosema	Lab required, targeted	[38,46,49]
Metagenomics	Episodic	Low	Multi-kingdom taxa (viruses, bacteria, fungi, plants)	High cost, bioinformatics	[14,98,99]
Digital (sensors)	Continuous (minutes)	None	Weight, Temperature, humidity, sound, CO_2_, flight activity	High cost, calibration	[9,12,55]
eDNA (honey/debris/pollen)	Episodic	Low	Pathogen DNA, floral DNA, pest DNA	No real-time output	[14,18,51,100]

**Table 2:** Multi-layer H-BEWS framework.

Layer	Data Sources	Key Signals	Biological Interpretation	References
**Digital**	Weight, T°, humidity, acoustics, CO_2_, flight counters, imaging	Weight loss/gain, thermal instability, acoustic changes, reduced flights, abnormal CO_2_	Colony activity, brood status, thermoregulation, swarming, queenlessness, foraging stress	[10,11,57,73,74]
**Molecular**	Honey eDNA, debris eDNA, pollen DNA	Pathogens (viruses, bacteria, fungi), pests (*Varroa*, *Aethina*), floral DNA	Disease pressure, pest invasion, forage resources, biodiversity	[14,18,53,104]
**Chemical**	Honey, wax, pollen, bee bread, hive air	Pesticides, acaricides, heavy metals, PFAS, VOCs	Contaminant exposure history, landscape chemical risk, air pollution	[30,116,135]
**Ecological**	Land use, weather, crop calendars, floral maps	Forage availability, land-use intensity, floral mismatch, climate stress	Nutritional stress, landscape context, exposure pathways	[112,150,151]

## Data Availability

Not applicable.

## Abbreviations

The following abbreviations are used in this manuscript:

ABPV (Acute Bee Paralysis Virus); AI (Artificial Intelligence); AMPA (Aminomethylphosphonic acid); AmSV-1 (*Apis mellifera Solinvivirus-1*); BQCV (Black Queen Cell Virus); CBPV (Chronic Bee Paralysis Virus); CNN (Convolutional Neural Network); CO_2_ (Carbon Dioxide); COI (Cytochrome c Oxidase Subunit I); CV (Computer Vision); DNA (Deoxyribonucleic Acid); DWV (Deformed Wing Virus); eDNA (Environmental DNA); EU (European Union); GBH (Glyphosate-Based Herbicide); GC-MS (Gas Chromatography–Mass Spectrometry); GIS (Geographic Information System); H-BEWS (Honeybee-Based Early Warning System); IoT (Internet of Things); ITS (Internal Transcribed Spacer); MIMS (Membrane Inlet Mass Spectrometry); MFCC (Mel-Frequency Cepstral Coefficients); ML (Machine Learning); mRNA (Messenger RNA); PFAS (Per- and Polyfluoroalkyl Substances); PFOS (Perfluorooctanesulfonic acid); PFOA (Perfluorooctanoic acid); PCR (Polymerase Chain Reaction); qPCR (Quantitative Polymerase Chain Reaction); RASFF (Rapid Alert System for Food and Feed); rbcL (Ribulose-1,5-bisphosphate carboxylase/oxygenase large subunit); RFID (Radio-Frequency Identification); RH (Relative Humidity); RNA (Ribonucleic Acid); RT-PCR (Reverse Transcription Polymerase Chain Reaction); SBV (Sacbrood Virus); T° (Temperature); TinyML (Tiny Machine Learning); trnL (Transfer RNA Leucine); VOC (Volatile Organic Compound); YOLO (You Only Look Once); 16S rRNA (16S Ribosomal RNA).
